# Magnetic Titanium Dioxide Nanocomposites as a Recyclable SERRS Substrate for the Ultrasensitive Detection of Histidine

**DOI:** 10.3390/molecules29122906

**Published:** 2024-06-19

**Authors:** Hailin Wen, Miao Li, Chao-Yang Zhao, Tao Xu, Shuang Fu, Huimin Sui, Cuiyan Han

**Affiliations:** School of Pharmacy, Qiqihar Medical University, Qiqihar 161000, Chinazhao33447@qmu.edu.cn (C.-Y.Z.); harvey-333@163.com (T.X.);

**Keywords:** SERRS, Fe_3_O_4_@TiO_2_, azo coupling, histidine, recycle

## Abstract

A highly sensitive, selective and recyclable histidine detection method based on magnetic Fe_3_O_4_@mTiO_2_ (M-TiO_2_) nanocomposites with SERRS was developed. Mesoporous M-TiO_2_ nanoparticles were functionalized with 4-aminothiophenol and then coupled with histidine through an azo coupling reaction in 5 min, producing the corresponding azo compound. The strong and specific SERRS response of the azo product allowed for ultrasensitive and selective detection for histidine with an M-TiO_2_ device loaded with Ag NPs due to the molecular resonance effect and plasmonic effect of Ag NPs under a 532 nm excitation laser. The sensitivity was further enhanced with the magnetic enrichment of M-TiO_2_. The limit of detection (LOD) was as low as 8.00 × 10^−12^ mol/L. The M-TiO_2_ demonstrated applicability towards histidine determination in human urine without any sample pretreatment. Additionally, the M-TiO_2_ device can be recycled for 3 cycles with the photodegradation of the azo product under UV irradiation due to TiO_2_-assisted and plasmon-enhanced photocatalysis. In summary, a multifunctional and recyclable M-TiO_2_ device was synthesized based on azo coupling and SERRS spectroscopy for ultra-sensitive and specific histidine sensing. In addition, the proposed system demonstrated the potential for the multiplex determination of toxic compounds in the fields of food safety, industrial production and environmental protection, which benefit from the fingerprint property and universality of SERRS.

## 1. Introduction

Amino acids are a type of organic acid that exist extensively in nature. They are the basic units composing proteins as well as the material foundation for life activity metabolism. Histidine plays a critical role in the active sites of some enzymes. In addition, it is involved in the regulation of the nervous system through histamine [1]. Histidine has the function of dilating blood vessels and lowering blood pressure; thus, it can be used in clinically treatment of angina pectoris, heart failure and coronary heart disease [2]. Many studies have shown that L-histidine contributes to the body’s physiological antioxidant capacity [3]. An abnormal level of histidine is an indicator for many related diseases. Therefore, it is of great significance to establish a simple, economical and sensitive method for histidine determination, which will become an important means of health diagnosis and disease screening and also serve as a reference for treatment and nutritional supplementation for various populations.

Currently, there are various methods for detecting histidine, including fluorescence methods [4,5,6,7,8,9,10,11,12,13,14], colorimetry method [15,16,17,18,19], electrochemistry [20], capillary electrophoresis (CE) [21], high-performance liquid chromatography (HPLC) [22,23], ultra-performance liquid chromatography-mass spectrometry (UPLC-MS) [24] and gas chromatography–mass spectrometry (GC-MS) [25,26]. These methods have some limitations in practical applications to some extent. UPLC-MS and GC-MS methods are costly due to specialized equipment and experienced operators. Colorimetry and CE methods offer rapid detection but often cannot achieve the required sensitivity. Derivatization processes and the use of environmentally unfriendly reagents are usually required for histidine detection with HPLC methods. For many electrochemical methods, complex electrode fabrication processes are inevitable, and they generally suffer from low selectivity. So far, large amounts of fluorescence-based probes have been developed for histidine because of their low cost, high sensitivity, high selectivity, simple pretreatment and rapid detection. Despite researchers having made great progress in the determination of histidine with fluorescence, fluorescence-based methods are still limited in utilizing quantum dots and small molecule dyes. Many probes suffer from the disadvantages, including challenging synthesis steps, heavy metal ions, high toxicity and low photostability. Tang et al. prepared a near-infrared fluorescence imprinted (CdTe@MIP) capillary sensor for the ultra-sensitive detection of histidine with an LOD of 0.08 pmol/L [8], while the linear range (0.1–1.8 pmol/L) was rather narrow. Additionally, optical microscopy has been applied for histidine based on sensor imaging with relatively long sample treatment time [27,28]. Liao et al. developed a DNAzyme-based liquid crystal biosensor for histidine with a label-free, none-amplified procedure according to changes of optical images with an LOD of 50 nmol/L [27]. Therefore, it is a vital issue to establish a simple, rapid, sensitive and economical method for histidine determination.

Surface-enhanced Raman spectroscopy (SERS) is a highly sensitive vibrational spectroscopic technique that provides molecular vibration-specific fingerprint information and allows for multiplex identifications. Furthermore, SERS enables rapid, non-destructive and in situ detection [29,30,31,32]. The SERS technique receives increasing interest in various fields, such as interface and surface science, physics, life sciences, art authentication and material science [33,34]. Vahid et al. [35] fabricated a SERS active substrate combining silver nanoparticles (NPs) and fluorine doped tin oxide for histidine detection. The detection limit was as low as 10^−9^ mol/L. Therefore, SERS provides a powerful tool for the ultra-sensitive analysis of histidine.

Our previous report had indicated that histidine could be detected through rapid azo coupling with 4-aminothiophenol (PATP) and SERRS spectroscopy with an LOD of 4.33 × 10^−11^ mol/L [36]. Strong Raman responses of histidine-derived azo products are generated, which benefit from its large Raman scattering cross section for a more sensitive determination of histidine indirectly.

Nowadays, the rapid development of SERS has driven the extension of SERS active substrates from noble metals to transition metals, semiconductor nanomaterials and metal–semiconductor composites. Bifunctional and even multifunctional SERS composite substrates simultaneously perform the functions of target recognition, optics, catalysis and magnetism, such as Fe_3_O_4_/Ag, Fe_3_O_4_/TiO_2_ and TiO_2_/Ag. We therefore formulated a design to combine magnetic nanomaterials with mesoporous TiO_2_ as a recyclable SERRS substrate for the rapid separation and the ultra-sensitive and selective detection of histidine.

In this paper, magnetic Fe_3_O_4_@mTiO_2_ (M-TiO_2_) nanocomposites with a mesoporous structure were synthesized and functionalized by PATP molecules for histidine capture through an azo coupling reaction and rapid separation from complex matrixes by an external magnet. The strong fingerprint SERRS responses of histidine-derived azo compounds by loading these nanodevices with Ag NPs enable the sensitive and selective quantitative determination of histidine. Additionally, the recyclability of the sensor was achieved with the degradation of histidine-derived azo compounds owing to TiO_2_-assisted and plasmon-enhanced photocatalysis. The sensor was applied to human urine samples successfully.

## 2. Results and Discussion

### 2.1. Characterization of M-TiO_2_

**Scheme 1:**
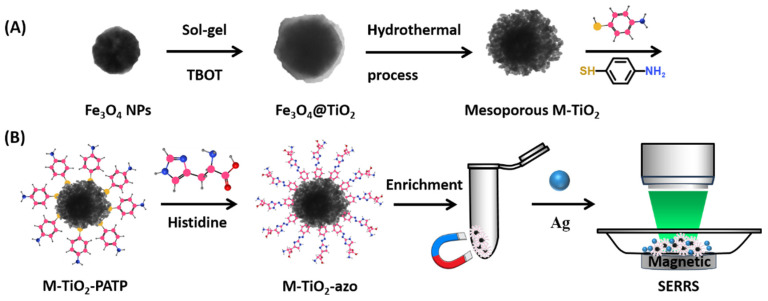
(**A**) Synthetic procedure for mesoporous M-TiO_2_ core/shell microspheres; (**B**) azo coupling process of M-TiO_2_ with histidine and SERRS-based detection.

**Figure 1:**
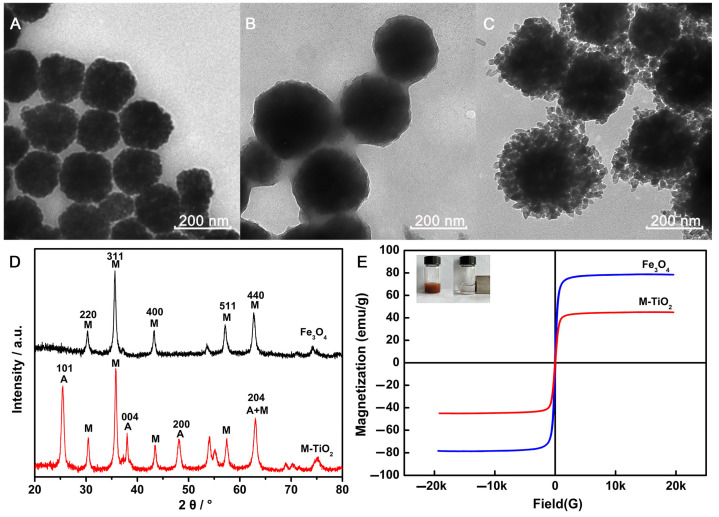
The TEM images of (**A**) Fe_3_O_4_, (**B**) Fe_3_O_4_@TiO_2_, (**C**) M-TiO_2_; (**D**) XRD patterns and (**E**) magnetic hysteresis curves for Fe_3_O_4_ and M-TiO_2_. Symbol M represents peaks attributed to Fe_3_O_4_, and symbol A represents peaks attributed to anatase TiO_2_. The inset was the magnetic separation behavior of M-TiO_2_ in the solution. M-TiO_2_ was prepared with 40:20 volume ratio of ethanol to water and 1 mL of NH_3_·H_2_O.

The synthesis protocol of M-TiO_2_ nanocomposites was illustrated in Scheme 1A. The black Fe_3_O_4_ NPs were synthesized with a solvothermal reaction [37] and had a spherical shape with an average diameter of about 200 nm, as shown in Figure 1A. The compact TiO_2_ layer with an amorphous structure was directly deposited on the Fe_3_O_4_ NPs surface by TBOT hydrolysis (Figure 1B). A hydrothermal treatment was conducted, leading to the formation of a mesoporous TiO_2_ shell with an anatase crystal structure. Thus, the surface area of TiO_2_ was increased, which was favorable for the adsorption and degradation of PATP. The average diameter of M-TiO_2_ was about 240 nm (Figure 1C), and the initial black Fe_3_O_4_ suspension turned brown. The images of the Fe_3_O_4_, Fe_3_O_4_@TiO_2_ and M-TiO_2_ prepared under different conditions were shown in Figure S1. The TiO_2_ shells and their crystalline phase were confirmed with X-ray diffraction (XRD). Compared with the XRD peaks of Fe_3_O_4_ (symbol M), peaks at 25.2°, 37.8° and 48.2° attributed to anatase TiO_2_ (symbol A) were observed (Figure 1D). The average microcrystalline size of TiO_2_ nanocrystals on M-TiO_2_ was about 16.2 nm, calculated by the peak at 25.2° according to the Scherrer formula [38]. The TEM image (Figure 1C) of M-TiO_2_ also confirmed the calculated results, indicating that the porous structure of M-TiO_2_ was composed of many TiO_2_ nanocrystals. Additionally, the chemical composition of the surface for M-TiO_2_ was characterized with a field emission transmission electron microscope–energy dispersive spectrometer (FETEM-EDS), elemental mapping (Figure S2).

The magnetic properties of Fe_3_O_4_ and M-TiO_2_ composite microspheres were investigated using a vibrating sample magnetometer (Figure 1E). The inset illustrated the magnetic separation behavior of M-TiO_2_ in the solution. The magnetic hysteresis curves of Fe_3_O_4_ and M-TiO_2_ showed the absence of significant coercivity and remanence, demonstrating that the microspheres were superparamagnetic. The saturation magnetization (Ms) value of Fe_3_O_4_ NPs reached 78.3 emu/g. Upon coating with an anatase-type TiO_2_ layer, the Ms value of M-TiO_2_ microspheres dropped to 44.7 emu/g. The TiO_2_ content of the composite microspheres was accordingly estimated to be 42.9 wt% by Ms value before and after TiO_2_ coating [39]. The high TiO_2_ content was responsible for the large number of PATP binding sites on M-TiO_2_ microspheres.

Notably, the solvent composition (ethanol and water) and concentration of NH_3_·H_2_O during hydrothermal treatment on amorphous TiO_2_ shells have great effects on the formation of M-TiO_2_ microsphere structure. We synthesized M-TiO_2_ NPs in different solvent mixtures (ethanol/water = 60:0, 40:20, 20:40 and 0:60, *v*/*v*) while the amount of NH_3_·H_2_O was maintained at 1 mL. The TEM results (Figure S3) indicated that the anatase TiO_2_ shells appeared to exhibit different surface morphology, and better porosity was achieved for TiO_2_ shells in a 40:20 volume ratio of ethanol to water. Then, NH_3_·H_2_O in different volumes (0, 1, 2, 3 mL) were added to prepare four M-TiO_2_ samples under the 40:20 volume ratio of ethanol to water. As shown in Figure S4, TiO_2_ layers exhibited a porous structure, even without NH_3_·H_2_O. When the volume of NH_3_·H_2_O exceeded 1 mL, TiO_2_ nanocrystals with sheet-like structures began to appear and bind to Fe_3_O_4_ NPs’ surface. Apparently, the morphology of TiO_2_ nanocrystals prepared with 1 mL NH_3_·H_2_O was more uniform and regular [39]. Therefore, M-TiO_2_ nanocomposites prepared in the 40:20 volume ratio of ethanol to water with 1 mL NH_3_·H_2_O were employed to subsequent functionalization and coupling. Moreover, M-TiO_2_ can be rapidly collected by an external magnet within 10 s (Figure 1E inset), indicating that the magnetic responsiveness of Fe_3_O_4_ was well preserved after TiO_2_ coating.

### 2.2. Azo Coupling of M-TiO_2_ with Histidine

The synthesized M-TiO_2_ was functionalized with PATP to capture histidine through an azo coupling reaction, as shown in Scheme 1B. The NH_2_ groups of PATP molecules adsorbed on the M-TiO_2_ surfaces were activated into corresponding diazonium ions. Aromatic diazonium ions react with active aromatic compounds (anilines, phenols or some heterocyclic substances) by electrophilic substitution, generating azo compounds with a N=N group [40]. In the present study, the coupling reaction between PATP-functionalized M-TiO_2_ (M-TiO_2_-PATP) and histidine can be completed within 5 min. By loading the M-TiO_2_-azo device with Ag NPs under a 532 nm excitation wavelength, histidine can be rapidly identified and detected as a result of the large Raman scattering cross section and SERRS fingerprint information of the corresponding azo product. In addition, the degradation of azo compounds and the recovery of nanomaterials were achieved with TiO_2_-assisted and plasmon-enhanced photocatalysis.

### 2.3. UV-Vis Absorption Spectra

Appropriate amounts of Fe_3_O_4_, M-TiO_2_, M-TiO_2_-PATP, M-TiO_2_-N≡N^+^, M-TiO_2_-blank and M-TiO_2_-azo were dispersed into ethanol for the UV-Vis absorption spectra characterization. In addition, the UV-Vis absorption spectra of M-TiO_2_ synthesized in different ethanol and water volume ratios were also obtained (Figure S5). As shown in Figure S6a,b, the intensity of the wide absorption peak around 650 nm increased after Fe_3_O_4_ NPs were coated with TiO_2_ nanocrystals. After coupling with histidine, a distinct absorption peak attributed to the formation of azo compounds appeared at approximately 550 nm. Considering a resonance effect [41] and the plasmon resonance of Ag NPs, a 532 nm laser was selected as the excitation source in the Raman test in order to achieve stronger Raman signals for ultrasensitive detection.

### 2.4. SERRS Spectra

For Raman measurements, all samples of M-TiO_2_ assemblies were gathered in a small area of an aluminum plate through an external magnet to promote the separation of M-TiO_2_ aggregation from the bulk solution and concentrate the target molecules for SERRS measurements. Thus, the interferences of other substances will be eliminated to a large extent, and the SERRS responses will be largely improved. In our study, a 532 nm laser line was employed as the excitation source, which afforded the optimal surface enhancement of Ag NPs and provided a strong resonance enhancement for histidine-derived azo products. We inferred that extremely high sensitivity can be achieved for the detection of histidine using M-TiO_2_-azo and a Ag NP sensing system under 532 nm. As shown in Figure S7, Ag NPs were adopted as the SERS substrate under 532 and 780 nm, respectively. Clearer and much stronger SERRS fingerprint vibration peaks were observed under 532 nm, especially 1389 and 1420 cm^−1^ due to the chromophore that was responsible for the absorption at around 550 nm. Adsorption at 780 nm is far away from the electronic transition energy of histidine-derived azo compounds, leading to the fact that the resonance effect cannot be produced between the laser and target molecule. Additionally, Ag NPs are unable to form strong plasmon resonance at 780 nm. Therefore, 532 nm was employed for the subsequent determination for histidine, which attributed to molecular resonance and plasmon enhancements.

We studied the histidine detection performance of M-TiO_2_ synthesized with different ethanol/water ratios. As shown in Figure S8, as the proportion of water in the system gradually increased, the SERRS performance of four M-TiO_2_-azo samples first increased and then gradually decreased. Among them, M-TiO_2_ synthesized with ethanol and water in 40:20 volume ratios, which had a more uniform porous structure (Figure 1C), was proven to possess optimal SERRS detection performance for histidine.

The pH is a crucial factor for an azo coupling reaction. Thus, we also explored the effect of Na_2_CO_3_ solution concentration in the coupling reaction on the performance of the M-TiO_2_ nanodevice for histidine detection. With the remaining conditions unchanged, the concentration of the Na_2_CO_3_ solution varied from 0% to 10% in the coupling reaction. The SERRS results showed (Figure S9) that the histidine-derived azo products obtained with 2% Na_2_CO_3_ had the maximum intensities of their characteristic peaks. Therefore, it was demonstrated that Na_2_CO_3_ with a concentration of 2% was more beneficial to the coupling reaction and was selected for the following histidine sensing by M-TiO_2_.

**Figure 2:**
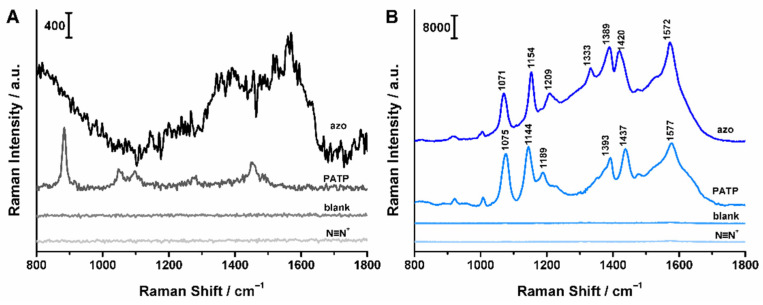
(**A**) The Raman spectra of M-TiO_2_-PATP, N≡N^+^, azo and blank assemblies and (**B**) the corresponding SER(R)S spectra by loading with Ag NPs. The azo product attached to M-TiO_2_ was derived from 10^−3^ mol/L histidine. Excitation wavelength: 532 nm.

PATP can be easily self-assembled onto the M-TiO_2_ surface due to the covalent binding of the sulfhydryl (-SH) functional group with TiO_2_ [42]. When PATP was attached to M-TiO_2_, weak Raman signals of PATP were observed (Figure 2). Immediately upon the addition of Ag NPs to the M-TiO_2_-PATP assembly, we detected a much stronger signal of 4,4′-dimercaptoazobenzene (DMAB) [43] owing to the SERS enhancement provided by the plasmonic Ag NPs. The SERS signals of DMAB at 1144, 1393 and 1437 cm^−1^ proved that PATP was successfully attached to M-TiO_2_. No obvious Raman signals appeared for M-TiO_2_-N≡N^+^ and blank samples, while very weak Raman peaks at 1300–1600 cm^−1^ for M-TiO_2_-azo were observed (Figure 2A). When we induced Ag NPs into the system, there were almost no SERS responses for M-TiO_2_-N≡N^+^ and blank samples so that they did not interfere with histidine detection. Significantly, SERRS spectra with abundant vibrational information were observed for M-TiO_2_-azo samples due to plasmon enhancement from Ag NPs. SERRS intensities of the peaks at 1389 and 1420 cm^−1^ were especially greatly enhanced. The peak at 1389 cm^−1^ corresponds to the C-H bending vibration and C-C stretching vibration on the benzene ring, while the peak at 1420 cm^−1^ represents the stretching vibration of trans -N=N- in the azo molecule, which was a characteristic feature of the azo product. The unique SERRS fingerprint and strong SERRS response of azo products attached to M-TiO_2_ makes it available for ultra-sensitive and specific determination of histidine.

In contrast to diazonium ions, azo products are stable at room temperature, which is crucial for SERRS measurements, while diazonium ions that do not react with histidine are unstable at room temperature and eventually form p-mercaptophenol [44]. Since the phenol group has a very weak affinity with Ag NPs [45], it is almost undetectable with SERS and therefore does not interfere with the SERRS analysis of azo compounds.

### 2.5. Sensitivity

**Figure 3:**
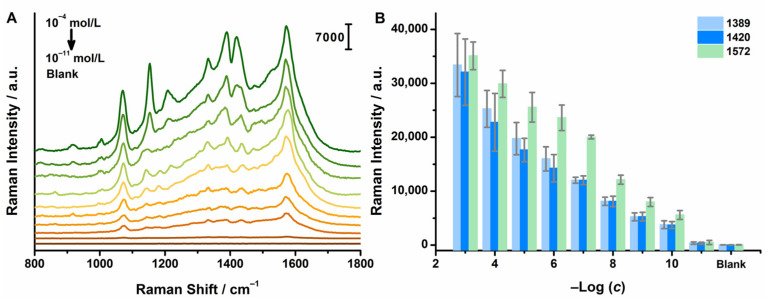
(**A**) The representative histidine concentration-dependent SERRS spectra of M-TiO_2_-azo loaded with Ag NPs and (**B**) their peak intensities at 1389, 1420 and 1572 cm^−1^. Excitation wavelength: 532 nm.

In order to explore the sensitivity of our M-TiO_2_ sensor for histidine, M-TiO_2_-PATP was coupled with different concentrations of histidine standard solutions, generating a series of histidine concentration-dependent M-TiO_2_-azo assemblies for their SERRS responses under the laser emission of 532 nm. The SERRS signals were first baseline corrected with NGS Labspec 5 and plotted with OriginPro 8.5. Representative concentration-dependent SERRS spectra and the plot of SERRS intensities (*I*_1389_, *I*_1420_, *I*_1572_) versus the negative logarithm of the histidine concentration were demonstrated in Figure 3. With the decrease in histidine concentration, the SERRS intensities of the characteristic peaks from histidine-derived azo products reduced gradually. Additionally, there exhibited good linear relationships between SERRS intensities (*I*_1389_, *I*_1420_, *I*_1572_) and the negative logarithm of the histidine concentration in the range of 10^−4^–10^−11^ mol/L, respectively. The linear equations were *I*_1389_ = 2897.247205logc + 32,202.921408 (R^2^ = 0.9892), *I*_1420_ = 2823.166185logc + 31,415.63635 (R^2^ = 0.9930) and *I*_1572_ = 4750.971325logc + 52,613.411661 (R^2^ = 0.9864). We also calculated the limits of detection (LODs), which were 8.29 × 10^−12^, 8.00 × 10^−12^ and 9.06 × 10^−12^ mol/L (3 *S*_B_/*m*), which are much lower than those of other methods previously reported. This is due to the strong plasmon resonance of Ag NPs and resonance effect under 532 nm excitation wavelength, which greatly improves the SERRS signal of histidine-derived azo products. Moreover, the enrichment of magnetic M-TiO_2_ nanomaterials by an external magnet also increases the sensitivity for histidine.

### 2.6. Recycling of M-TiO_2_ Sensing Device for Histidine

**Figure 4:**
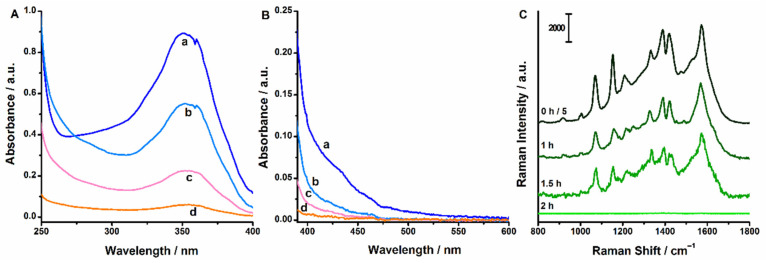
(**A**) The UV-Vis spectra of histidine (1 mg/mL)-derived azo product without M-TiO_2_ (a) and mixed with M-TiO_2_ after exposure to UV irradiation for 1 h (b), 2 h (c) and 3 h (d) ranging from 250–400 nm and (**B**) 390–600 nm; (**C**) The SERRS spectra of M-TiO_2_-azo (derived from 10^−3^ mol/L histidine) after exposure to UV irradiation loaded with Ag NPs under 532 nm. The SERRS spectral intensity of M-TiO_2_-azo irradiated by 0 h was divided by 5.

**Figure 5:**
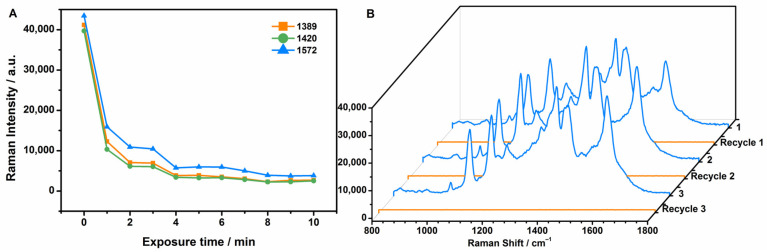
(**A**) SERRS intensities at 1389, 1420 and 1572 cm^−1^ of histidine (10^−3^ mol/L)-derived azo product attached to M-TiO_2_ loaded with Ag NPs as a function of exposure time under 532 nm; (**B**) The recyclability of the M-TiO_2_ nanodevice for histidine.

Azo compounds can be degraded with TiO_2_-assisted photocatalysis through UV light irradiation [46]. In order to investigate the photocatalysis capacity of the present M-TiO_2_ nanocomposites, azo product solution derived from 1 mg/mL histidine was mixed with M-TiO_2_ nanocomposites and irradiated with a 312 nm UV lamp (12.0 W). Here, azo-attached M-TiO_2_ was not directly used since its high background was unfavorable to UV-Vis spectra measurements. After UV irradiation, the M-TiO_2_ nanocomposites were separated by a magnet, and the supernatants were measured with UV-Vis absorption spectroscopy. UV irradiation time-dependent UV-Vis spectra were shown in Figure 4A,B. It can be seen that the characteristic absorption peaks at 352 and 433 nm were almost invisible after 3 h UV irradiation, indicating the decomposition of azo products. Additionally, azo-attached M-TiO_2_ nanodevices prepared with 10^−3^ mol/L histidine after exposure to UV irradiation were directly characterized by loading with Ag NPs for SERRS measurements (Figure 4C). As the UV irradiation time increases, the SERRS responses decrease progressively to zero due to the decomposition of azo products. Furthermore, we found that the SERRS intensities of histidine-derived azo compounds attached to M-TiO_2_ loaded with Ag NPs decrease dramatically with the increasing of laser exposure time under the laser emission of 532 nm (Figure 5A). After 1 min exposure to the 532 nm laser, the intensities of characteristic bands from azo products reduced by 75% rapidly and then decreased gradually in 10 min. It is inferred that the photodegradation efficiency is increased due to the enhanced electric field at the TiO_2_/azo interface when M-TiO_2_-azo are brought in close contact with Ag NPs [47]. Therefore, it is suggested that our M-TiO_2_ nanocomposites can be re-used for histidine sensing with UV-cleaning. The re-functionalization of PATP to M-TiO_2_ and subsequent histidine-derived azo products were characterized by SERRS in Figure S11. Highly similar SERRS spectra were obtained to those in Figure 2. The recyclability of the M-TiO_2_ sensing device following the same procedure was displayed in Figure 5B. The complete recovery of SERRS signals was observed in up to three cycles, indicating that M-TiO_2_ can be re-functionalized with PATP and recycled for histidine sensing at least three times. The results demonstrated that the M-TiO_2_ nanodevice is a sustainable sensor for histidine, which makes it attractive as a SERRS substrate.

### 2.7. Interference Study

**Figure 6:**
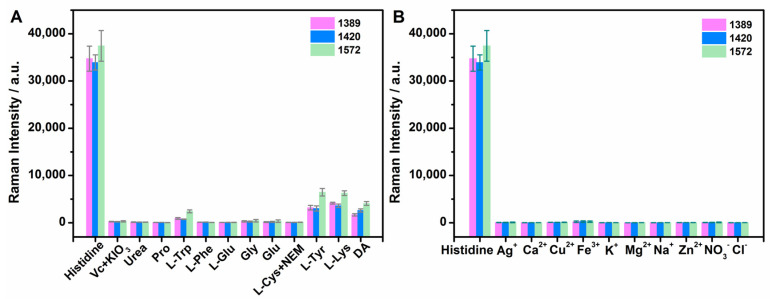
(**A**) SER(R)S intensities at 1389, 1420 and 1572 cm^−1^ of M-TiO_2_ towards 10^−3^ mol/L of histidine, potential co-existing substances in biological fluids, including (**A**) Vc, urea, Pro, L-Trp, L-Phe, L-Glu, Gly, Glu, L-Cys, L-Tyr, L-Lys, DA, (**B**) Ag^+^, Ca^2+^, Cu^2+^, Fe^3+^, K^+^, Mg^2+^, Na^+^, Zn^2+^, NO_3_^−^ and Cl^−^. The corresponding concentrations of the interferences were 10^−2^, 4, 10^−2^, 10^−2^, 10^−2^, 10^−2^, 10^−2^, 10^−1^, 10^−3^, 10^−3^, 10^−3^, 10^−3^, 10^−2^, 1, 10^−2^, 1, 4, 1, 4, 1, 4 and 4 mol/L, respectively. Excitation wavelength: 532 nm.

To explore the applicability of our method for detecting histidine in biological samples, interference studies towards potential co-existing substances were investigated, including ascorbic acid (Vc), urea, proline (Pro), L-tryptophan (L-Trp), L-phenylalanine (L-Phe), L-glutamic (L-Glu), glycine (Gly), glucose (Glu), L-cysteine hydrochloride (L-Cys), L-tyrosine (L-Tyr), L-lysine (L-Lys), dopamine (DA), Ag^+^, Ca^2+^, Cu^2+^, Fe^3+^, K^+^, Mg^2+^, Na^+^, Zn^2+^, NO_3_^−^ and Cl^−^. The concentrations of the above interference substances were 10^−2^, 4, 10^−2^, 10^−2^, 10^−2^, 10^−2^, 10^−2^, 10^−1^, 10^−3^, 10^−3^, 10^−3^, 10^−3^, 10^−2^, 1, 10^−2^, 1, 4, 1, 4, 1, 4 and 4 mol/L, respectively. As shown in Figure 6, the interference samples exhibit weak signals compared with 10^−3^ mol/L histidine. Additionally, N-ethylmaleimide (NEM) was introduced into the system to eliminate the interference of L-Cys. KIO_3_ was used to oxidize Vc. Thus, the selectivity can be preliminarily proved for histidine determination in biological fluids.

### 2.8. Accuracy and Precision

The high sensitivity and selectivity of the proposed approach make the determination of histidine in trace levels attainable, even in complex biological samples. The proposed sensor was employed with histidine in human urine. Recovery tests were conducted to verify the accuracy and precision with the standard addition method for human urine samples. Histidine standard solutions were added into blank human urine and detected with our method. Three parallel samples were prepared for each concentration. The linear equation fitted with a peak intensity at 1420 cm^−1^ was used for the calculation of the recovery rate. As shown in Table 1, the recoveries were between 95.0% and 102.3%, and the relative standard deviations were within 8.1%, proving the high accuracy and reproducibility of the methodology. As a consequence, our M-TiO_2_ based SERRS assay for histidine has definite potentiality in complex systems with simplicity, rapidness and reliability.

### 2.9. Comparison with Other Methods for Histidine

The experimental results indicate that our proposed detection method based on M-TiO_2_ exhibits high sensitivity for histidine detection, with satisfactory levels of repeatability and accuracy. In comparison to other methods for histidine, our method offers relatively high sensitivity over a wide range of concentrations and a simple sample treatment process (Table 2). In addition, the detection system was improved compared with our previous report [36] about SERRS-based assays for histidine in terms of sensitivity and recyclability due to the magnetic enrichment and photocatalysis of M-TiO_2_ nanocomposites. The LOD has been reduced 10 times. Therefore, the M-TiO_2_-based SERRS assay for histidine has the superiority of simplicity and rapidness in the coupling process and urine sample treatments, ultra-high sensitivity and selectivity and nanomaterial recyclability.

### 2.10. Universality

In our study, histidine was determined with a M-TiO_2_ SERRS sensor based on an azo coupling reaction. The NH_2_ groups of PATP molecules adsorbed on the M-TiO_2_ surfaces were activated into corresponding diazonium ions. Aromatic diazonium ions can also react with other anilines, phenols or some heterocyclic substances by electrophilic substitution, generating corresponding azo products with a N=N group [40], which have unique SERRS spectra for specific recognition and determination. Distinguishable SERRS signals of bisphenol A and estradiol with our sensor were shown in Figure S11. Thus, our approach offers a universal sensing system for the multiplex determination of toxic compounds.

## 3. Materials and Methods

### 3.1. Chemical Reagents

Iron chloride hexahydrate (FeCl_3_·6H_2_O), silver nitrate, PATP (≥98%) and gold chloride trihydrate (99%) were purchased from Shanghai Aladdin Biochemical Technology Co., Ltd. (Shanghai, China) Titanium(IV) butoxide (≥97%) was purchased from Sigma-Aldrich Chemical Co., Ltd. (St. Louis, MO, USA). L-histidine, ascorbic acid (Vc), urea, proline (Pro), L-tryptophan (L-Trp), L-phenylalanine (L-Phe), L-glutamic (L-Glu), glycine (Gly), glucose (Glu), L-cysteine hydrochloride (L-Cys), L-tyrosine (L-Tyr), L-lysine (L-Lys) and dopamine (DA) were purchased from Beijing Dingguo Changsheng Biotechnology Co., Ltd. (Beijing, China). Hydrochloric acid, ammonia water (25%) was obtained from Shenyang Esat China Reagent Plant (Shenyang, China). Sodium acetate anhydrous, ethylene glycol, ethanol, sodium nitrite and sodium carbonate anhydrous, sodium chloride, potassium chloride, calcium chloride, magnesium chloride, zinc chloride, potassium iodate and copper sulfate were obtained from Tianjin Kaitong Chemical Reagent Co., Ltd. (Tianjin, China). Acetonitrile and trisodium citrate dihydrate were purchased from Tianjin Fuchen Chemical Reagent Co., Ltd. (Tianjin, China). The purified water purchased from Wahaha Group Co., Ltd. (Hangzhou, China) was used throughout the experiment.

### 3.2. Apparatus and Measurement

Raman spectra were measured with the Thermo Scientific DXR3xi Raman Imaging Microscope (Thermo Scientific, Waltham, MA, USA) equipped with an excitation laser wavelength of 532 nm (power: 10.0 mW) and 780 nm (power: 24.0 mW). Raman equipment was wavelength calibrated automatically every 30 days and used with an electron multiplied charge-coupled device (EM-CCD) system at 23 °C. All Raman spectra were recorded with a 50× microscope objective. The laser power, exposure time and scanning times were 1.0 mW, 0.05 s and 50 repetitions, respectively. The SEERS responses were tested by mixing 20 μL M-TiO_2_-azo with 20 μL Ag NPs. The mixtures were loaded into an aluminum plate, then concentrated and fixed by an external magnet under the plate for Raman measurements. Transmission electron microscopy (TEM) images were taken on a Hitachi-HT7700 transmission electron microscope (Hitachi, Tokyo, Japan) at an accelerating voltage of 200 kV. Field emission transmission electron microscope–energy dispersive spectrometer (FETEM-EDS) characterization was conducted on a JEOL JEM-2100F transmission electron microscope (JEOL Ltd., Tokyo, Japan) at an accelerating voltage of 200 kV. Magnetic characterization was performed with a vibrating sample magnetometer (model: 7404, LakeShore, Carson, CA, USA) at 300 K. XRD patterns were collected on an X’Pert Pro (Panalytical, Almelo, The Netherlands) diffraction meter with Cu KR radiation at λ = 0.154 nm operating at 40 kV and 40 mA. UV-Vis absorption spectra were characterized with UV-Vis spectrophotometer (model: T6 New Century, AliExpress, Hangzhou, China) in the range of 200–800 nm.

### 3.3. Synthesis of M-TiO_2_ Nanocomposites

M-TiO_2_ NPs were synthesized according to a previously reported method [40]. In brief, 0.81 g of ferric chloride hexahydrate and 2.61 g of anhydrous sodium acetate were dissolved in 30 mL ethylene glycol and sonicated for 5 min. The suspension was transferred to a Teflon-lined stainless steel high-pressure reactor, which was then heated to 200 °C and held for 8 h. After that, the reactor was naturally cooled to room temperature. The Fe_3_O_4_ black particles in the suspension were collected by an external magnet and then washed three times with ethanol in a certain volume. The resulting Fe_3_O_4_ particles were vacuum dried at 50 °C for 6 h.

The compact TiO_2_ layer was directly deposited on the Fe_3_O_4_ surface using tetrabutyl orthotitanate (TBOT) hydrolysis. An amount of 50 mg of the Fe_3_O_4_ particles obtained above were ultrasonically dispersed in a mixture of 90 mL ethanol, 30 mL acetonitrile and 0.5 mL NH_3_·H_2_O. Then, 1 mL TBOT was added into the suspension under mechanical stirring for 1.5 h. After that, Fe_3_O_4_@TiO_2_ particles were collected and washed three times with ethanol.

The amorphous TiO_2_ shells were transformed into anatase TiO_2_ through a hydrothermal process. An amount of 50 mg of Fe_3_O_4_@TiO_2_ particles were dispersed in a 60 mL solution system with ethanol and water (40:20, *v*/*v*). An amount of 1 mL NH_3_·H_2_O was added to the suspension. The mixture was heated at 160 °C for 20 h in a Teflon-lined stainless steel high-pressure reactor. After the reactor was naturally cooled to room temperature, M-TiO_2_ nanocomposites in brown were separated by an external magnet. Then, the particles were washed three times with ethanol and re-dispersed in 10 mL ethanol.

### 3.4. Functionalization of M-TiO_2_ and Azo Coupling

An amount of 1 mL M-TiO_2_ nanocomposites were dispersed in 100 mL ethanol solution containing 2 × 10^−3^ mol/L PATP with mechanical stirring for 2 h, then separated and washed with an external magnet. The PATP-functionalized M-TiO_2_ (M-TiO_2_-PATP) were activated into diazonium ions (M-TiO_2_-N≡N^+^) by NaNO_2_ (10 mL, 5%) and HCl (10 mL, 0.1 mol/L) in an ice water bath for 10 min. M-TiO_2_-N≡N^+^ were separated and mixed with Na_2_CO_3_ (20 mL, 2%) and histidine (10 mL, 10^−3^ mol/L) in the ice-water bath under mechanical stirring for 5 min, thus histidine was captured by M-TiO_2_-N≡N^+^ based on azo coupling reaction, generating histidine-derived azo product (M-TiO_2_-azo). The same coupling processes were performed on M-TiO_2_-N≡N^+^ without target histidine to obtain the M-TiO_2_-blank sample.

### 3.5. Preparation of Ag NPs

Ag NPs were prepared according to the commonly used Lee–Meisel method [48]. An amount of 0.036 g of silver nitrate were dissolved in 200 mL water under constant stirring. An amount of 4 mL of 1% sodium citrate were immediately added into silver nitrate solution in slight boiling state. The resulting Ag NPs in gray green with the average size of 48.36 ± 10.11 nm (Figure S12) were naturally cooled to room temperature and stored in a refrigerator at 4 °C.

### 3.6. Preparation of Histidine Standard Solutions

The standard solutions were prepared using a stepwise dilution method. First, histidine (19.1 mg) was accurately weighed and dissolved in 10 mL water (with the addition of a small amount of HCl for enhancing solubility through ultrasonic treatment) to obtain a standard stock solution with the concentration of 10^−2^ mol/L. Subsequently, the stock solution was further diluted by water to generate a series of standard solutions in different concentrations, which were stored at 4 °C prior to utilization. NEM with a final concentration of 10^−2^ mol/L was added to eliminate the effects of L-Cys. KIO_3_ was added to oxidize Vc to prevent the interference.

### 3.7. Preparation of Urine Samples

First of all, 10 mL human urine from a healthy volunteer was centrifuged at 7000 rpm for 10 min to obtain the supernatant as blank urine sample. The urine samples were prepared with standard addition method. Briefly, a certain volume of stock solution was added into urine. Then, the samples were centrifuged, obtaining urine samples in certain concentrations. All these samples were stored at 4 °C and diluted by 10,000 times for detection.

## 4. Conclusions

In summary, mesoporous TiO_2_-coated magnetic Fe_3_O_4_ nanocomposites were synthesized for histidine sensing with SERRS spectroscopy. PATP-functionalized M-TiO_2_ can capture histidine through an azo coupling reaction in 5 min, generating corresponding azo products. The strong and unique SERRS response of azo products provided the possibility of the ultrasensitive and selective determination of histidine upon the plasmon enhancement of Ag NPs and resonance enhancement. Sensitivity can be further improved due to the separation and concentration of M-TiO_2_-azo composites by an external magnet. The LOD of the method was as low as 8.00 × 10^−12^ mol/L with a linear range of 10^−4^–10^−11^ mol/L. The proposed M-TiO_2_ nanocomposites were applied to histidine detection with good accuracy and precision in human urine samples treated only with centrifugation. Moreover, the target can be degraded due to TiO_2_-assisted and plasmon-enhanced photocatalysis. M-TiO_2_ nanocomposites can be recycled at least three times. In addition, the proposed M-TiO_2_ devices can be expected for the multiplex determination of toxic compounds (anilines or phenols) by specific vibrational fingerprints. Thus, we present recyclable M-TiO_2_ devices for the sensitive and selective SERRS sensing of histidine, which offer a promising platform for toxicants evaluation and supervision in the fields of food safety, industrial production and environmental protection.

## Figures and Tables

**Table 1:** Results of recovery tests for histidine determination in human urine.

Found without Spiking (mol/L)	Spiked (mol/L)	Found (mol/L)	Recovery (%)	RSD (%)
	1.80 × 10^−2^	1.86 × 10^−2^	102.3	8.1
1.82 × 10^−4^	1.80 × 10^−3^	1.73 × 10^−3^	95.0	5.3
	1.80 × 10^−4^	3.55 × 10^−4^	98.1	4.2

**Table 2:** A comparison of the proposed method with other methods for histidine detection.

Materials	Methods	Samples	Sample Treatment Time	Instrument Test Time	Linear Range	LOD (nmol/L)	Reference
NMM/G-4-Cu(II)	Fluorescence	Urine	2 h 40 min	/	0.003–10.0 μmol/L	3	[4]
Probes: SDO, SDM and SDP with Ag^+^	Fluorescence	Standard	/	/	/	3840, 2430, 6640	[6]
PFBT PDs/Cu^2+^	Fluorescence	Blood serum	25 min	/	0.1–920 μmol/L	79.6	[7]
CdTe@MIP capillary	Fluorescence	Serum, urine	15 min	/	0.1–1.8 pmol/L	8 × 10^−5^	[8]
DNAzyme & HCR & Triplex formation	Fluorescence	Urine	10 h	/	5.7–455 nmol/L	2.0	[9]
Au/Cu NCs	Fluorescence	Serum, urine	/	/	3–10,000 nmol/L	0.9	[10]
Cu-CDs	Fluorescence	Serum, urine	Serum: 11 min. Urine: /	/	0.1–15 μmol/L	30	[11]
CuNCs	Fluorescence	Urine	20 min	/	0.05–40.0 μmol/L	1.6	[12]
DNA-Ag NCs	Fluorescence	Urine	225 min	/	0–8 μmol/L	96	[13]
N, S-CDs/Fe^3+^	Dual fluorescence/ colorimetry	Serum, urine	Serum: 25 min. Urine: /	/	0.1–3.0, 100–375 μmol/L	30, 24,200	[14]
G-quadruplex-Cu(II) metalloenzyme	Colorimetry	Standard	2 h 4 min	/	0.01–1 μmol/L	10	[15]
Indicator (murexide)-displacement assay (IDA)	Colorimetry	Urine	22 min	/	2–30 μmol/L	400	[17]
Lysine-functionalized Ag NPs	Colorimetry	Standard	10 min	/	5.0–30.0 μmol/L	5000	[18]
Au NPs, aptamer	Colorimetry	Serum	10 min	/	0–400 nmol/L	3.6	[19]
GNPs-GNSs, aptamer	Electrochemistry	Standard	/	30 s	10 pmol/L–10 μmol/L	10^−4^	[20]
Ru(bpy)_3_^2+^/TPA	CE-ECL	Standard	/	7 min	5–35, 35–150 μmol/L	10^3^	[21]
Post-column derivatization with *o*-phthalaldehyde	HPLC	Saliva	10 min	/	0.5–5.0 μmol/L	50	[22]
Pre-column AQC derivatization	UPLC-MS	Plasma, urine	10 min 20 s	8 min for chromatography	2–2000 μmol/L	310	[24]
Pre-column AQC derivatization	GC-MS	Herbal raw materials	27 h 60 s	/	1–100 μg/mL	30	[25]
DNAzyme-based LC biosensor	Optical image	Standard	1 h	/	/	50	[27]
Au NPs-CuAAC	Dark-field microscopy	Serum	36 min	/	5.0–80 μmol/L	2100	[28]
M-TiO_2_ nanocomposites	SERRS	Urine	15 min	/	10^−4^–10^−11^ mol/L	8.00 × 10^−3^	This work

## Data Availability

The data presented in this study are available in article and Supplementary Materials.

## Supplementary Materials

The following supporting information can be downloaded at https://www.mdpi.com/article/10.3390/molecules29122906/s1, Figure S1: Images of nanocomposites; Figures S2–S4: TEM and FETEM-EDS image of nanocomposites; Figures S5 and S6: UV-Vis spectra of the synthesized M-TiO_2_ nanocomposites; Figure S7: SER(R)S spectra of M-TiO_2_-azo under 532 and 785 nm; Figure S8: SERRS spectra of M-TiO_2_-azo synthesized with different ethanol/water ratios; Figure S9: Sodium carbonate concentration-dependent SERRS spectra of M-TiO_2_-azo; Figure S10: Raman spectra of recycling process; Figure S11: SERRS spectra of M-TiO_2_-azo derived from bisphenol A, estradiol and histidine; Figure S12: The TEM image and diameter distribution of the prepared Ag NPs.
