# Supplementary material for: Magnetic Titanium Dioxide Nanocomposites as a Recyclable SERRS Substrate for the Ultrasensitive Detection of Histidine
Source: Molecules. 2024 Jun 19;29(12):2906. doi: 10.3390/molecules29122906 (PMC11206314; doi:10.3390/molecules29122906)
Supplement: Supplementary file 1 [file molecules-29-02906-s001.zip › molecules-3042337-SI.pdf]

<u>Supporting Information for Publication</u>

Magnetic Titanium Dioxide Nanocomposites as a Recyclable SERRS Substrate for the Ultrasensitive Detection of Histidine

Hailin Wen, Miao Li, Chao-Yang Zhao, Tao Xu, Shuang Fu, Huimin Sui * and Cuiyan Han *

School of Pharmacy, Qiqihar Medical University, Qiqihar 161042, China; zhao33447@qmu.edu.cn (C.-Y.Z.); harvey-333@163.com (T.X.)

* Correspondence: suihm_9@163.com (H.S.); hcymuphar@qmu.edu.cn (C.H.)

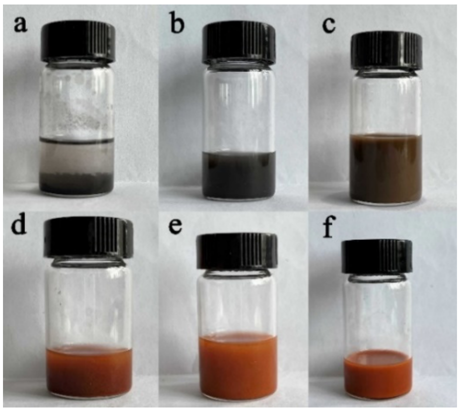

Figure S1 Images of (a) Fe_3O_4 and (b)Fe_3O_4@TiO_2 and M-TiO_2, prepared under different ethanol/water ratios of (c) 60:0, (d) 40:20, (e)20:40, and (f) 0:60.

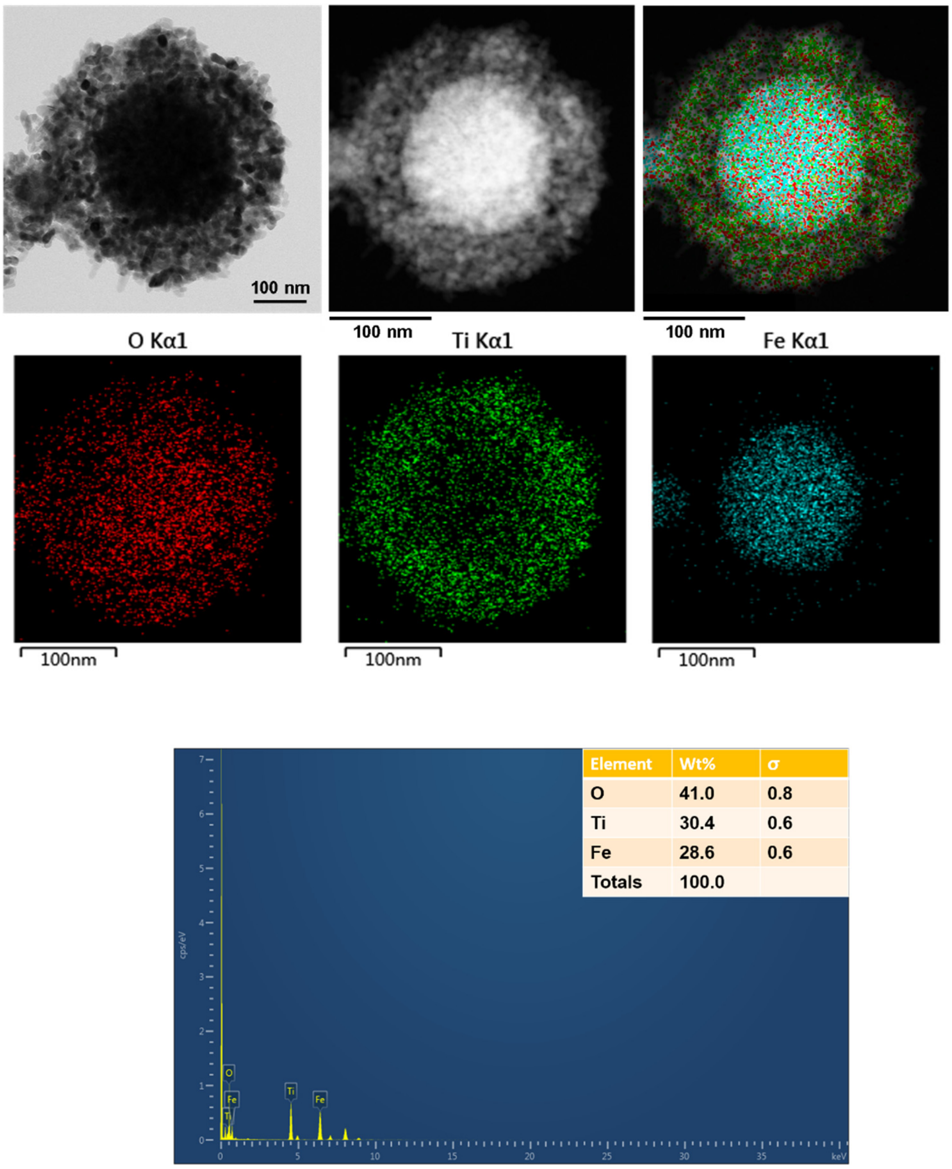

Figure S2 Field emission transmission electron microscope-energy dispersive spectrometer (FETEM-EDS) results of the prepared M-TiO_2 nanocomposites.

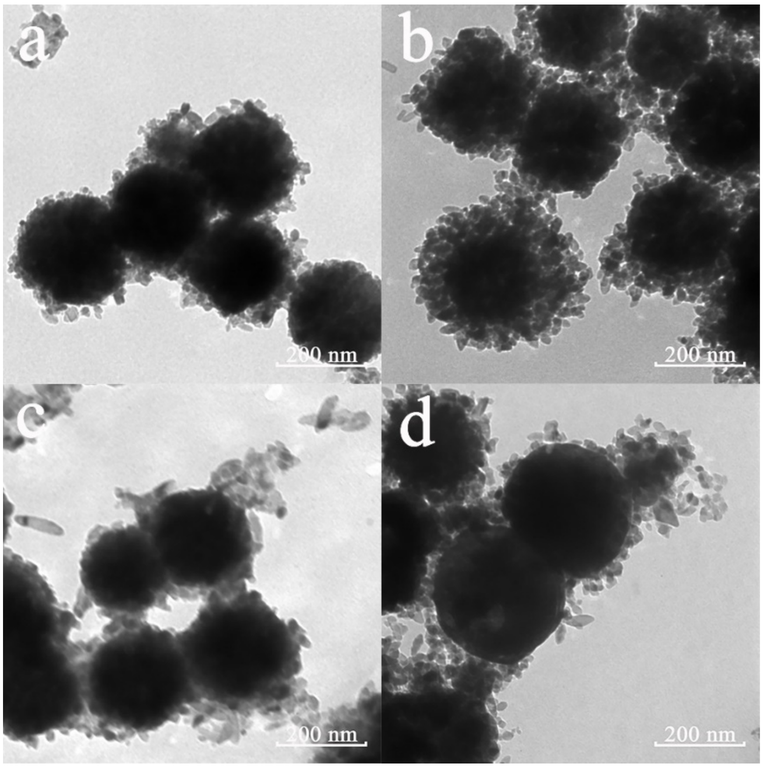

Figure S3 TEM images of M-TiO_2 prepared in different ethanol/deionized water ratios: (a) 60:0; (b) 40:20; (c) 20:40; (d) 0:60. The volume of $NH_3 \cdot H_2O$ is 1 mL.

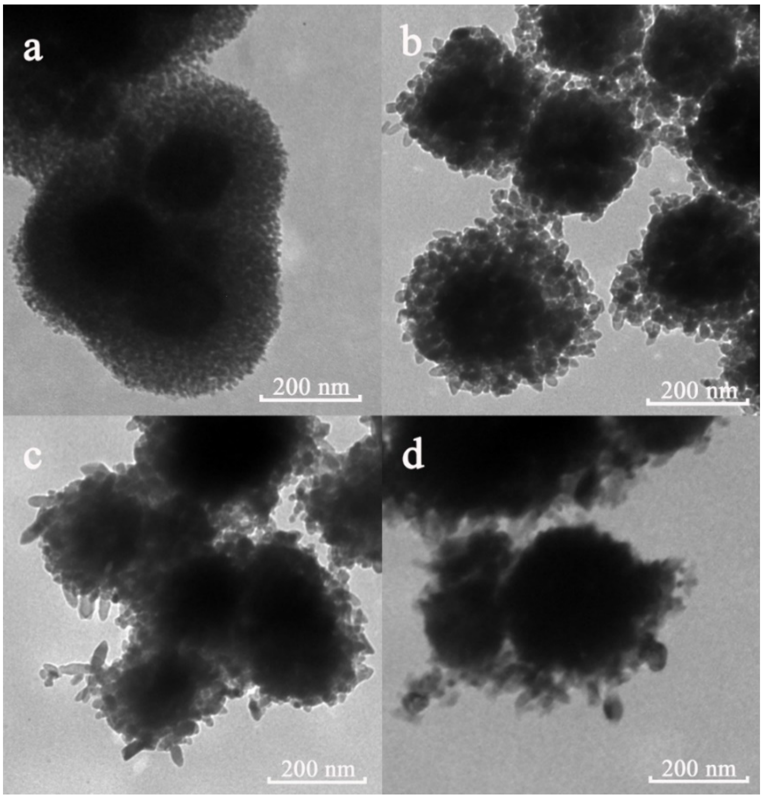

Figure S4 TEM images of M-TiO_2 prepared with different amounts of $NH_3 \cdot H_2O$: (a) 0, (b) 1, (c) 2 and (d) 3 mL. The volume ratio of ethanol to water is 40:20.

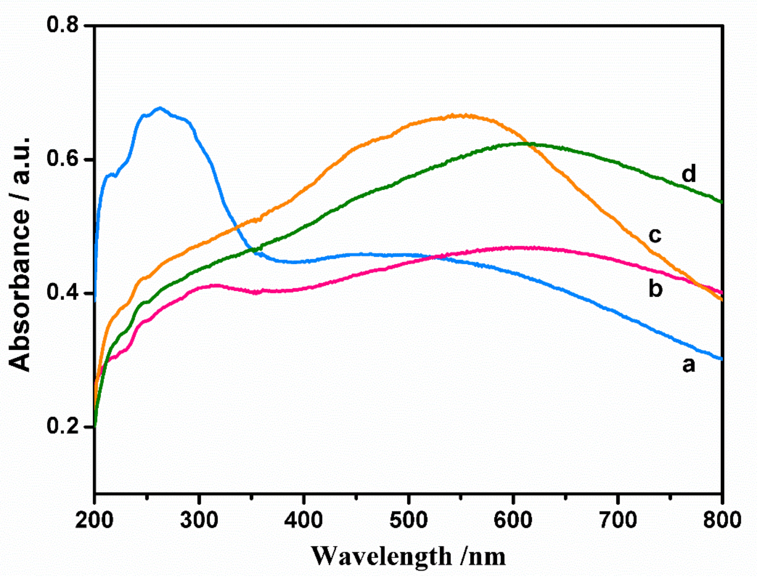

Figure S5 UV-Vis spectra of the M-TiO_2 synthesized in different ethanol/water ratios of (a) 60:0, (b) 40:20, (c)20:40, and (d) 0:60.

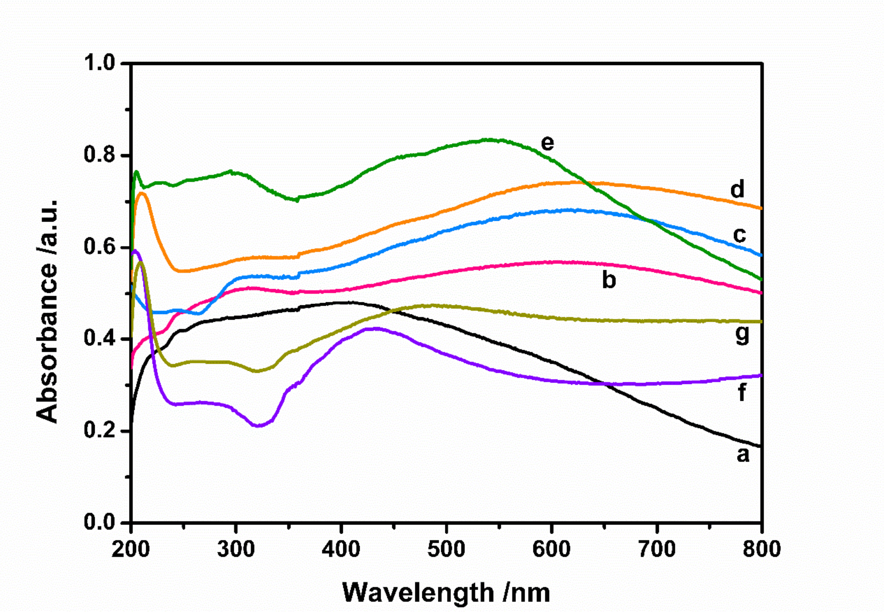

Figure S6 UV-Vis spectra of the synthesized nanocomposites: (a) Fe_3O_4; (b) M-TiO_2; (c) M-TiO_2-PATP; (d) M-TiO_2-$N{\equiv}N^+$; (e) M-TiO_2-azo; (f) M-TiO_2-azo mixed with Ag NPs; (g) M-TiO_2-blank.

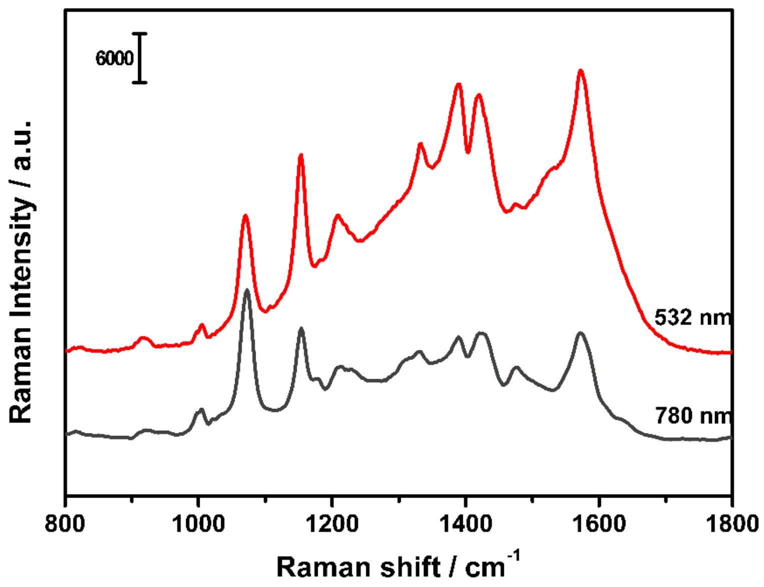

Figure S7 SER(R)S spectra of M-TiO_2-azo loaded with AgNPs under 532 and 785 nm excitation sources.

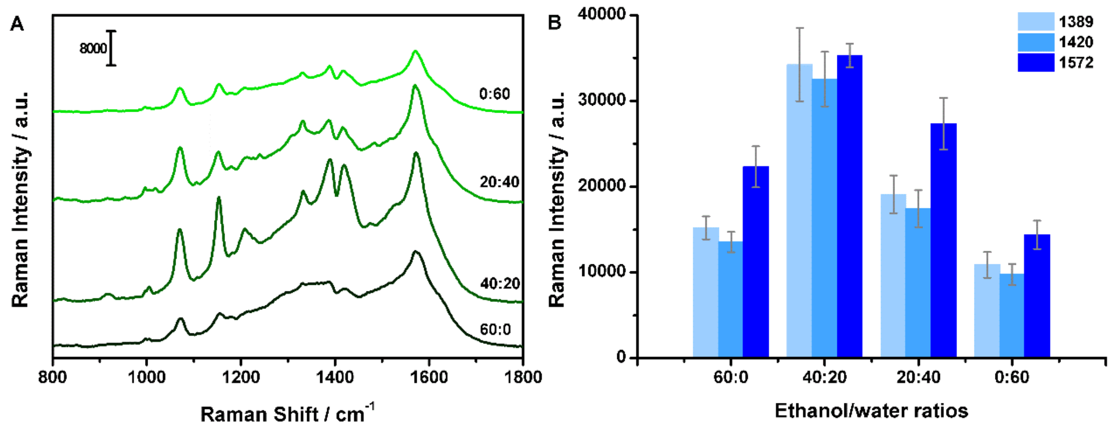

Figure S8 SERRS spectra (A) and SERRS intensities of characteristic peaks (B) of histidine-derived azo compound by M-TiO_2 synthesized with different ethanol/water ratios. The SERRS intensity of M-TiO_2-azo (40:20) was divided by 3.

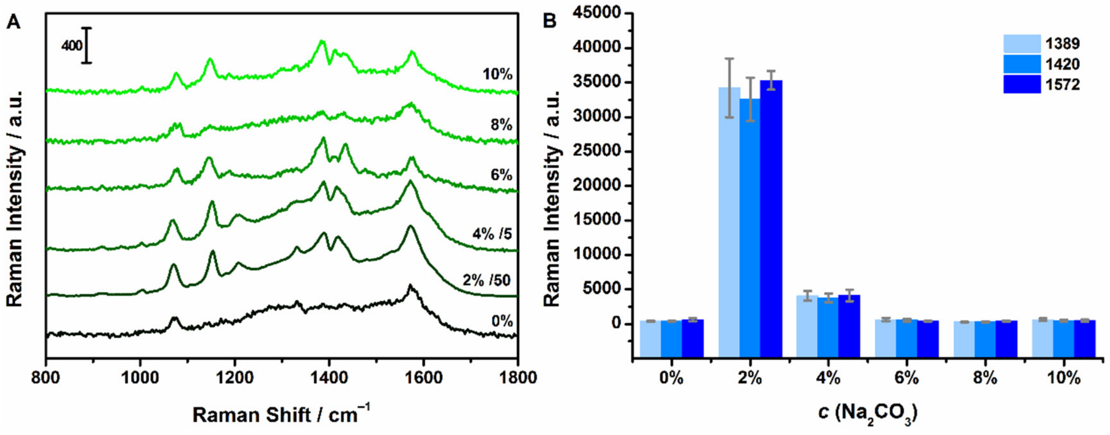

Figure S9 Sodium carbonate concentration-dependent SERRS spectra (A) and SERRS intensities of characteristic peaks (B) of M-TiO_2-azo loaded with Ag NPs. Excitation wavelength: 532 nm. The SERRS spectral intensities of M-TiO_2-azo prepared with 2% and 4% Na_2CO_3 were divided by 50 and 5, respectively.

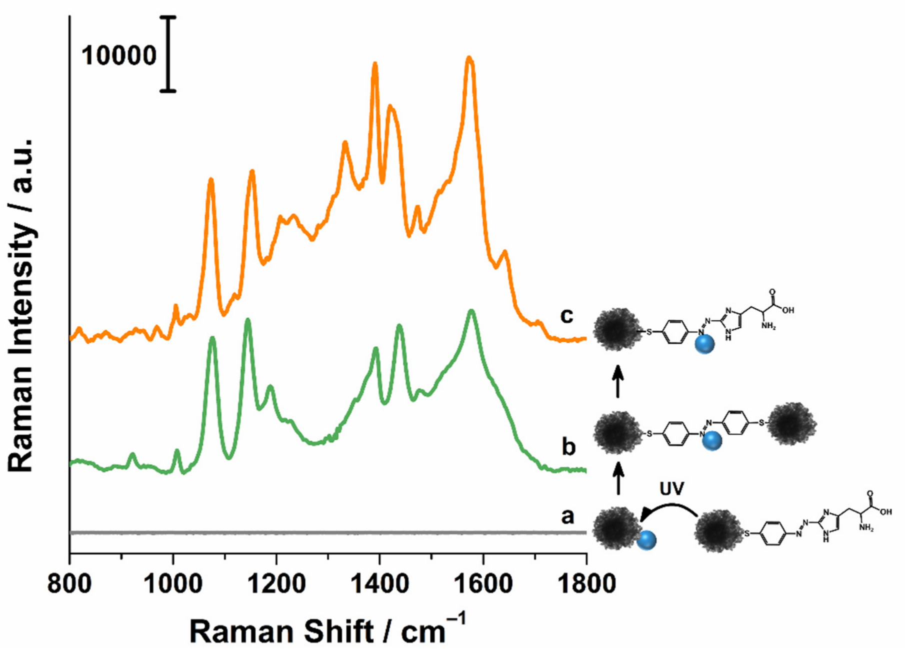

Figure S10 Raman spectra of M-TiO_2 attached with azo product after 3 h UV irradiation (a, with Ag NPs), and SERRS spectra of PATP re-functionalization (b) and histidine-derived azo (c) attached to the recycled M-TiO_2. Excitation wavelength: 532 nm.

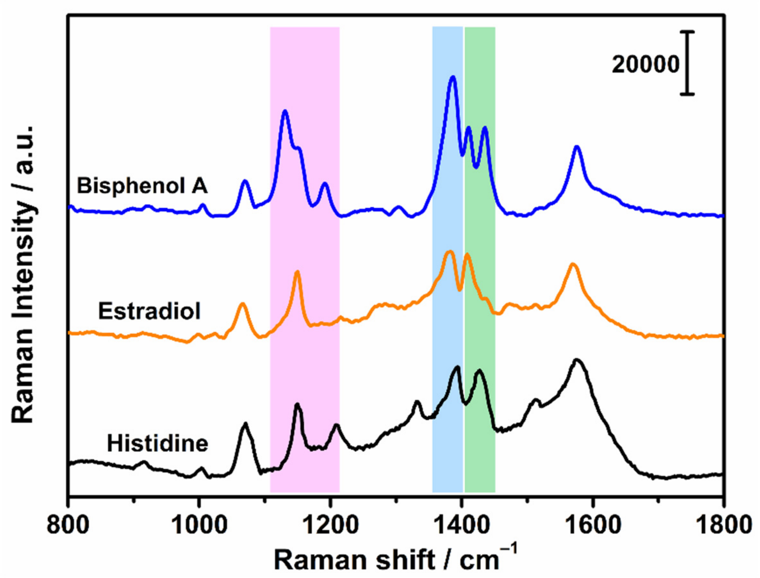

Figure S11 SERRS spectra of M-TiO_2-azo derived from bisphenol A, estradiol and histidine.

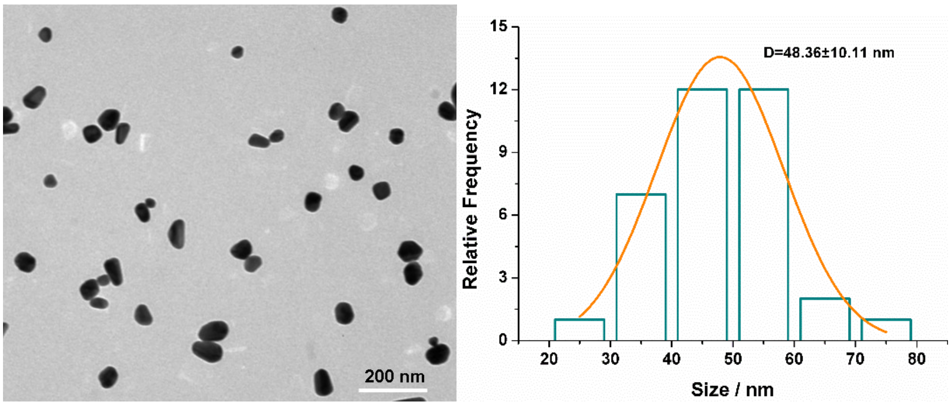

Figure S12 TEM image and diameter distribution of the prepared Ag NPs.
